# Impact of type 2 diabetes mellitus on the prognosis of patients with hepatocellular carcinoma after laparoscopic liver resection: A multicenter retrospective study

**DOI:** 10.3389/fonc.2022.979434

**Published:** 2022-12-15

**Authors:** Shi-Ye Yang, Mao-Lin Yan, Jin-Kai Feng, Yun-Fei Duan, Jia-Zhou Ye, Zong-Han Liu, Lei Guo, Jie Xue, Jie Shi, Wan Yee Lau, Shu-Qun Cheng, Wei-Xing Guo

**Affiliations:** ^1^ Department of Hepatic Surgery VI, Eastern Hepatobiliary Surgery Hospital, Second Military Medical University, Shanghai, China; ^2^ Department of Hepatobiliary and Pancreatic Surgery, Fujian Provincial Hospital, The Shengli Clinical Medical College of Fujian Medical University, Fuzhou, China; ^3^ Department of Hepatobiliary and Pancreatic Surgery, The Third Affiliated Hospital of Soochow University (Changzhou People’s Hospital), Jiangsu, China; ^4^ Department of Hepatobiliary Pancreatic Surgery, Affiliated Tumor Hospital of Guangxi Medical University, Guangxi, China; ^5^ Faculty of Medicine, The Chinese University of Hong Kong, Shatin, New Territories, Hong Kong, Hong Kong SAR, China

**Keywords:** laparoscopic liver resection (LLR), hepatocellular carcinoma (HCC), overall survival (OS), type 2 diabetes mellitus (T2DM), recurrence-free survival (RFS)

## Abstract

**Background:**

The effect of type 2 diabetes mellitus (T2DM) on survival of patients with hepatocellular carcinoma (HCC) after laparoscopic liver resection (LLR) has not been reported. This study aimed to explore the relationship between preoperative T2DM and long-term prognosis in HCC patients undergoing LLR.

**Methods:**

HCC patients receiving LLR as initial treatment at four cancer centers were retrospectively included in this study. Clinicopathological factors associated with the prognosis of HCC patients were identified using univariate and multivariate Cox regression analysis. Recurrence-free survival (RFS) and overall survival (OS) curves between different cohorts of patients were generated using the Kaplan-Meier method and compared using the log-rank test.

**Results:**

Of 402 HCC patients included, 62 patients had T2DM and 340 patients did not have T2DM. The OS and RFS of patients with T2DM were significantly worse compared to those without T2DM (*P* = 0.001 and 0.032, respectively). In Cox multivariate analysis, T2DM was identified as an independent risk factors for OS (HR = 2.31, 95% CI = 1.38–3.85, *P* = 0.001) and RFS (HR = 1.66, 95% CI = 1.08–2.55, *P* = 0.020).

**Conclusions:**

Following laparoscopic surgical approach, HCC patients with T2DM had poorer prognoses than those without T2DM. Preoperative T2DM was an independent risk factor for HCC patients. Thus, patients with concurrent HCC and T2DM should be closely monitored after LLR.

## Introduction

Hepatocellular carcinoma (HCC) is the sixth most common malignancy and the fourth leading cause of cancer-related mortality worldwide (1). Despite advancement in diagnostic and therapeutic techniques, the incidence of HCC is still on the rise due to the prevalence of hepatitis virus infection in the endemic Asia-Pacific region (2). In the West, non-alcoholic fatty liver disease (NAFLD) is becoming the most rapid growing etiology of HCC (3, 4). At present, hepatectomy is without a doubt the best curative treatment modality for patients with resectable HCC and well-preserved liver function (5). However, due to the high incidence of HCC recurrence, the long-term survival for patients with HCC after surgery is far from satisfactory. Prior studies suggested that up to 60%–70% of patients will relapse within 5 years following initial surgery (6, 7).

Type 2 diabetes mellitus (T2DM) is a common metabolic-associated disease, which is characterized by chronic hyperglycemia resulting from impaired insulin secretion, insulin resistance or a combination of both. It is an expanding global health problem and has reached epidemic proportions worldwide (8). Epidemiological studies revealed that T2DM is highly prevalent in HCC patients and increases the incidence of HCC among individuals with chronic hepatitis B or C virus infection (9–11). T2DM is also an established risk factor for the rapid progression of NAFLD to cirrhosis or HCC (12–14). Therefore, it is not uncommon to encounter patients who have concomitant HCC and T2DM in clinical practice.

With the popularization of minimally invasive surgery, laparoscopic liver resection (LLR) has been widely performed in recent years, and gradually becomes one of the main hepatectomy approach. Two propensity score matching (PSM) studies showed that LLR is as safe and effective as open liver resection (OLR) for HCC patients (15, 16). In previous OLR series, pre-existing T2DM was reported to have direct negative impact on short-term postoperative outcomes and long-term prognosis (17, 18). Wang et al (19). found that T2DM was associated with significantly lower overall survival (OS) in HCC patients after curative hepatectomy, especially in cases with cirrhosis. Choi et al. (20). documented that T2DM was correlated with intrahepatic HCC recurrence following surgical resection. However, there is currently no clear answer as to whether T2DM increases postoperative complication rates and worsens survival outcomes in HCC patients undergoing LLR.

In the present study, we aimed to investigate the effect of preoperative T2DM on postoperative outcomes for HCC patients undergoing laparoscopic hepatectomy using a multicenter database. Additionally, our previous study first reported T2DM was an independent risk factor for the incidence of microvascular invasion (MVI) in HBV-related HCC after OLR. Our study validated the association between T2DM and MVI using a cohort of HCC patients who underwent laparoscopic resection.

## Materials and methods

### Study design and patient selection

This retrospective study was conducted on consecutive HCC patients who underwent LLR as initial treatment between January 2016 and February 2018 at four hospitals, the Eastern Hepatobiliary Surgery Hospital (EHBH), Fujian Provincial Hospital (FPH), Changzhou People’s Hospital (CZPH), and Affiliated Tumor Hospital of Guangxi Medical University (ATHGMU). The preoperative clinical indicators and postoperative pathological results were retrieved by reviewing the data from electronic medical records. These patients were divided into the diabetic and non-diabetic groups according to whether they had preoperative T2DM. Laparoscopic operations were all performed by skilled surgeons who had passed the learning curves for LLR in the four participant centers.

The present study was performed according to the Declaration of Helsinki (as revised in 2013) and was approved by the Institutional Ethics Committees of EHBH, FPH, CZPH, and ATHGMU. Informed consent was obtained from all patients for their data to be used for research purposes. All types of personal identification on files were de-identified using surrogate numbers to secure patient privacy.

### Inclusion and exclusion criteria

Patients fulfilling the following criteria were eligible for enrollment: (I) age between 18 and 75 years; (II) Barcelona Clinic Liver Cancer (BCLC) stages 0–B HCC; (II) no previous anti-cancer treatment prior to LLR; (III) no history of other malignancies or major abdominal surgery; (IV) well-preserved liver function of Child-Pugh scores ≤ 7. The following exclusion criteria were predetermined: (I) incomplete demographic, serological, pathological, or follow-up data; (II) a preoperative diagnosis of T1DM; (III) conversion to open hepatectomy during LLR.

### Diagnostic standard of HCC, MVI and T2DM

The clinical diagnostic criteria for HCC were in line with the AASLD guidelines (21), and classified using the BCLC staging system (22). MVI was defined as tumor invasion of small blood vessels with endothelial cell linings that can only be detected under microscope (23). Generally, MVI was diagnosed by histopathological examinations based on hematoxylin eosin (HE)-stained slices. In cases whose diagnosis was uncertain, special immunohistochemical staining was performed (23, 24).

For new-onset T2DM, the definition of T2DM was in accordance with the diagnostic criteria of the American Diabetes Association: (I) diabetes symptoms and a random plasma glucose level ≥ 11.1 mmol/L; (II) a fasting plasma glucose (FPG) level ≥ 7.0 mmol/L; (III) 2-h plasma glucose level after a 75-g oral glucose tolerance test (OGTT) ≥ 11.1 mmol/L according to the WHO requirement; or (IV) the need for hypoglycemic medicines to control glucose levels (25). For long-standing T2DM, documented medical records were reviewed for the diagnosis.

### Procedure of laparoscopic liver resection and perioperative management

Patients with fasting plasma glucose level above 300 mg/dl were not candidates for LLR. To achieve good glycemic control in diabetic HCC patients preoperatively, long-acting insulin analogue and/or oral hypoglycemic drugs were administered, aiming to keep fasting plasma glucose level within 110 to 180 mg/dl and urinary glucose to less than 3 g/day. If metformin was prescribed, it was withdrawn before surgery to avoid lactic acidosis due to possible postoperative renal dysfunction (26).

The procedure of LLR was performed under general anesthesia as previously described (16, 27). The patient was placed in a supine position or a 30-degree reverse Trendelenburg position. A 12-mm peri-umbilical camera port was placed under direct vision, and 4 additional trocars were manipulated by the surgeon or assistants. Carbon dioxide pneumoperitoneum pressure was created and maintained at 12 mmHg. Intraoperative ultrasonography (IOUS) was routinely used to detect the location of target lesions and guide the resection planes. The resection line was predetermined using monopolar electrocautery. The intermittent Pringle’s maneuver was routinely applied to occlude the blood inflow of liver. The liver parenchyma was transected with a harmonic scalpel (Ethicon, Somerville, NJ, USA), and LigaSure (ValleyLab, Inc.). Small hepatic vessels were secured by endoclips or Hem-o-lok clips. The large vessels were divided by vascular staplers. Fibrin glue sealant was applied to the cut surface of the liver; and hemostasis was achieved using bipolar electrocautery. The resection area was meticulously checked for biliary leakage and bleeding. After LLR was completed, the resected specimen was placed in a retrieval bag and extracted through an enlarged incision site.

After surgery, insulin or oral hypoglycemic drugs were continuously used to maintain the serum glucose level under 200 mg/dl with monitoring of the blood and urinary sugar levels.

### Definitions

OS was measured from the date of LLR to the date of death or the end of this study. RFS was defined as the time between the dates of LLR and the date on which HCC recurrence was first diagnosed or the last contact of patients. Anatomical resection was defined as a complete removal of a tumor-bearding portal territory; otherwise, it was defined as non-anatomical resection. Major liver resection was defined as resection of 3 or more Couinaud liver segments; otherwise, it was defined as a minor hepatectomy. Surgical resection margin was defined as the shortest measured distance from the edge of the tumor to the plane of liver transection. Surgical resection margin was categorized as wide and narrow resection margin based on a cutoff of 1 cm (28, 29). For LLR, it was considered resection adequacy if the resection margin was more than 1 cm. Operative time was defined as the duration from the first incision to the final dressing being placed on the patient. Postoperative liver failure referred to a serum total bilirubin level exceeding 50 μmol/L and prothrombin time lower than 50% on or after postoperative day 5 (30). The definition of bile leakage was based on the criteria of the International Study Group of Liver Surgery (31).

### Follow-up

After discharge from hospital, all patients were followed up once every two months within the first year, and once every three months thereafter, until death or dropout from the follow-up program. Follow-up investigations included complete blood counts, liver and renal function, plasma glucose level, coagulation profiles, serum alpha-fetoprotein (AFP), and abdominal ultrasonography (US). Tumor recurrence was diagnosed by dynamic computed tomography (CT) and/or contrast-enhanced magnetic resonance imaging (MRI). Recurrent HCC was aggressively managed by repeated surgery, transarterial chemoembolization (TACE), radiofrequency ablation (RFA), molecular targeted therapy or immunotherapy according to the status of HCC and liver function of patients.

### Outcome measures

The primary endpoints were recurrence-free survival (RFS) and overall survival (OS). The secondary endpoints were surgery-related parameters, postoperative length of hospital stay and complications.

### Statistical analysis

Continuous variables were described as mean with standard deviation or median with interquartile range, and compared using the Student’s *t* test or Mann-Whitney *U* test. Categorical variables were compared using the χ^2^ or Fisher’s exact tests as appropriate. Univariate analysis and multivariate Cox regression analysis were applied to identify the independent risk factors for OS and RFS. After univariate analysis, only the significant variables (*P* < 0.05) were subjected to the multivariate Cox regression analysis by a backward stepwise selection method. Survival curves were delineated using the Kaplan–Meier method and compared by means of the log rank test. Moreover, univariate and multivariate logistic regression analyses were performed to detect the independent predictive factors for the incidence of MVI.

All statistical tests were two-sided, and *P* value < 0.05 was considered to indicate statistical significance. The statistical analyses were performed using SPSS version 26.0 (SPSS, Chicago, IL, USA) and R version 4.1.0 (R foundation for Statistical Computing, Vienna, Austria).

## Results

### Baseline characteristics of all HCC patients

**Figure 1:**
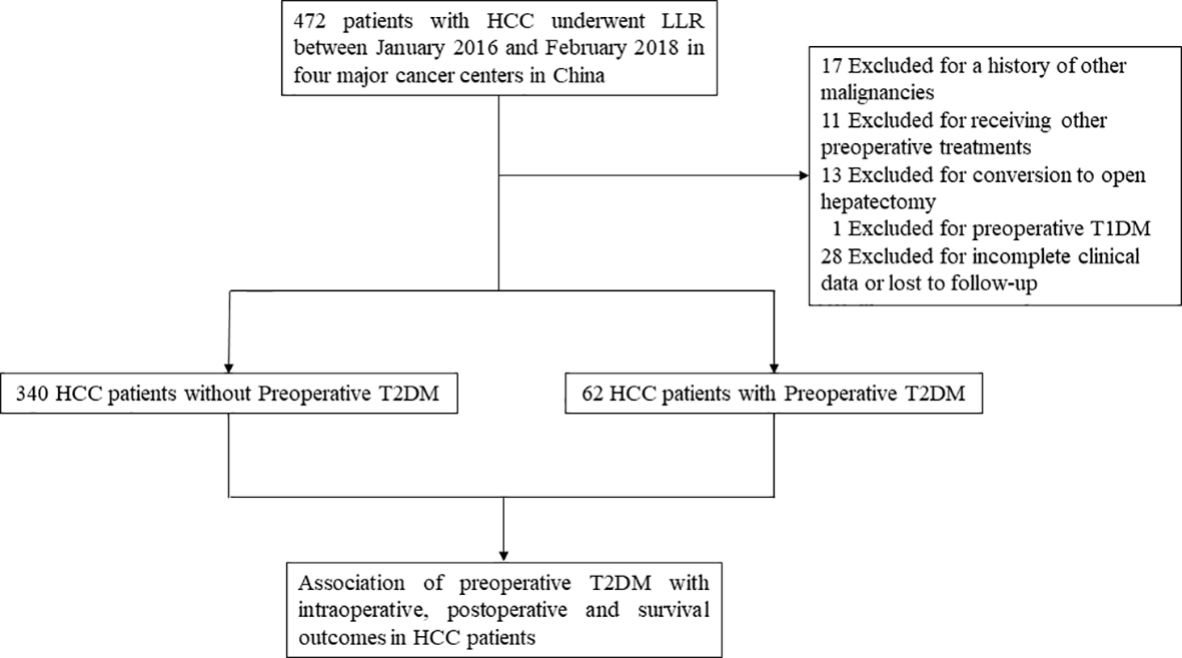
Study design frame and patient selection process. HCC, hepatocellular carcinoma; LLR, laparoscopic liver resection; T1DM, type 1 diabetes mellitus; T2DM, type 2 diabetes mellitus.

As shown in **Figure 1**, 472 HCC patients underwent LLR during the study period at the 4 hospitals. After excluding patients who were not eligible for this study, 402 HCC patients were enrolled. Among them, 62 were diabetic and 340 patients were non-diabetic. Compared to patients without T2DM, patients with preoperative T2DM were significantly older (*P* < 0.001), had significantly lower HBV-DNA load (*P* = 0.001), had markedly higher fasting plasma glucose level (*P* < 0.001), had markedly higher serum creatinine level (*P* = 0.005), and substantially higher proportion of MVI (*P* = 0.04). The other clinicopathological features were comparable between the T2DM and non-T2DM groups (**Table 1**).

**Table 1:** The baseline clinicopathological features of all HCC patients (n = 402).

Variables	Without T2DM (n=340)	With T2DM (n=62)	*P*
Age, years	56.0 (48.0-64.0)	63.0 (52.2-70.8)	<0.001
Sex			0.139
Female	53 (15.6%)	15 (24.2%)	
Male	287 (84.4%)	47 (75.8%)	
HBeAg			0.797
Negative	266 (78.2%)	47 (75.8%)	
Positive	74 (21.8%)	15 (24.2%)	
HBV DNA load, IU/mL			0.001
≤10^4^	62 (18.2%)	23 (37.1%)	
>10^4^	278 (81.8%)	39 (62.9%)	
Antiviral therapy			0.699
No	224 (65.9%)	43 (69.4%)	
Yes	116 (34.1%)	19 (30.6%)	
AFP, ng/mL			0.484
<400	208 (61.2%)	35 (56.5%)	
≥400	132 (38.8%)	27 (43.5%)	
ALT, U/L			0.776
≤44	243 (71.5%)	46 (74.2%)	
>44	97 (28.5%)	16 (25.8%)	
AST, U/L			0.821
≤44	261 (76.8%)	49 (79.0%)	
>44	79 (23.2%)	13 (21.0%)	
TBil, μmol/L			0.176
<17.1	258 (75.9%)	42 (67.7%)	
≥17.1	82 (24.1%)	20 (32.3%)	
ALB, g/L			1.000
<35	27 (7.94%)	5 (8.06%)	
≥35	313 (92.1%)	57 (91.9%)	
PT, s			0.547
≤13	131 (38.5%)	27 (43.5%)	
>13	209 (61.5%)	35 (56.5%)	
PLT, 10^9^/L			0.992
≤100	35 (10.3%)	7 (11.3%)	
>100	305 (89.7%)	55 (88.7%)	
Glucose, mmol/L			<0.001
≤7	328 (96.5%)	36 (58.1%)	
>7	12 (3.53%)	26 (41.9%)	
Creatinine, mg/dL			0.005
≤1.2	334 (98.2%)	56 (90.3%)	
>1.2	6 (1.76%)	6 (9.68%)	
WBC, 10^6^/L			1.000
<4000	36 (10.6%)	6 (9.68%)	
≥4000	304 (89.4%)	56 (90.3%)	
RBC, 10^12^/L	4.66 (4.26-5.01)	4.52 (4.20-4.97)	0.552
HGB, g/L			0.345
≤110	16 (4.71%)	5 (8.06%)	
>110	324 (95.3%)	57 (91.9%)	
Child-Pugh class			0.904
A	298 (87.6%)	54 (87.1%)	
B	42 (12.4%)	8 (12.9%)	
BCLC stage			0.971
0-A	246 (72.4%)	45 (72.6%)	
B	94 (27.6%)	17 (27.4%)	
Varices			0.701
Absent	328 (96.5%)	61 (98.4%)	
Present	12 (3.53%)	1 (1.61%)	
Tumor diameter, cm			0.164
≤5	218 (64.1%)	46 (74.2%)	
>5	122 (35.9%)	16 (25.8%)	
Tumor number			0.612
Solitary	296 (87.1%)	56 (90.3%)	
Multiple	44 (12.9%)	6 (9.68%)	
Tumor capsule			0.155
Complete	71 (20.9%)	18 (29.0%)	
Incomplete	269 (79.1%)	44 (71.0%)	
MVI			0.040
No	161 (47.4%)	20 (32.3%)	
Yes	179 (52.6%)	42 (67.7%)	
Cirrhosis			1.000
No	100 (29.4%)	18 (29.0%)	
Yes	240 (70.6%)	44 (71.0%)	

T2DM, type 2 diabetes mellitus; HBeAg, hepatitis B e antigen; HBV, hepatitis B virus; AFP, alpha-fetoprotein; ALT, alanine aminotransferase; AST, aspartate transaminase; TBil, total bilirubin; ALB, albumin; BCLC, Barcelona Clinic Liver Cancer; PT, prothrombin time; RBC, red blood cells; WBC, white blood cells; HGB, Hemoglobin; PLT: platelets; MVI, microvascular invasion.

### Independent prognostic factors analysis

As shown in **Table 2**, HBeAg (*P* = 0.029), preoperative T2DM (*P* = 0.001), hemoglobin level (*P* = 0.006), cirrhosis (*P* = 0.006), tumor diameter (*P* < 0.001), and MVI (*P* = 0.001) were independent prognostic factors of OS. Pre-existing T2DM (*P* = 0.02), cirrhosis (*P* = 0.01), tumor diameter (*P* < 0.001), tumor number (*P* = 0.007), and MVI (*P* = 0.045) were independent prognostic factors of RFS.

**Table 2:** Univariate analysis and multivariate Cox analysis of overall survival and recurrence free survival for all HCC patients (n=402).

Characteristics	Overall survival						Recurrence-free survival					
	Univariate analysis			Multivariate analysis			Univariate analysis			Multivariate analysis		
	HR	95% CI	*P* value	HR	95% CI	*P* value	HR	95% CI	*P* value	HR	95% CI	*P* value
Age (years)	1.01	(0.99-1.03)	0.286				1.00	(0.99-1.02)	0.671			
Sex (male vs female)	1.42	(0.75-2.68)	0.278				1.42	(0.88-2.30)	0.156			
HBeAg (positive vs negative)	1.82	(1.14-2.90)	0.012	1.70	(1.05-2.73)	0.029	1.06	(0.71-1.59)	0.766			
HBV DNA load (>10^4^ vs ≤10^4^) (IU/mL)	1.23	(0.69-2.18)	0.484				1.11	(0.75-1.67)	0.590			
Antiviral therapy (yes vs no)	0.82	(0.52-1.28)	0.387				0.90	(0.63-1.28)	0.555			
T2DM (yes vs no)	2.32	(1.41-3.81)	<0.001	2.31	(1.38-3.85)	0.001	1.55	(1.02-2.36)	0.040	1.66	(1.08-2.55)	0.020
RBC (10^12^/L)	0.93	(0.65-1.32)	0.683				1.01	(0.76-1.33)	0.963			
WBC (≥4000 vs <4000) (10^6^/L)	0.70	(0.36-1.36)	0.293				1.04	(0.60-1.80)	0.892			
HGB (>110 vs ≤110) (g/L)	0.34	(0.17-0.69)	0.003	0.37	(0.18-0.75)	0.006	0.50	(0.27-0.92)	0.026	0.73	(0.51-1.05)	0.087
PLT (>100 vs ≤100) (10^9^/L)	0.59	(0.32-1.09)	0.092				0.94	(0.55-1.61)	0.820			
TBil (≥17.1 vs <17.1) (μmol/L)	1.45	(1.00-2.09)	0.051				1.40	(0.97-2.0)	0.074			
ALB (≥35 vs <35) (g/L)	0.54	(0.27-1.09)	0.086				0.62	(0.36-1.06)	0.079			
AST (>44 vs ≤44) (U/L)	1.47	(0.91-2.38)	0.117				1.39	(0.96-2.02)	0.080			
ALT (>44 vs ≤44) (U/L)	1.05	(0.65-1.72)	0.836				1.14	(0.78-1.67)	0.496			
PT (>13 vs ≤13) (s)	1.43	(0.91-2.27)	0.124				1.25	(0.89-1.77)	0.198			
Glucose (>7 vs ≤7) (mmol/L)	2.07	(1.12-3.83)	0.020	1.55	(0.71-3.40)	0.274	1.05	(0.59-1.85)	0.878			
Creatinine (>1.2 vs ≤1.2) (mg/dL)	1.65	(0.52-5.23)	0.398				1.92	(0.49-7.69)	0.352			
Varices (Absent vs Present)	1.35	(0.43-4.28)	0.611				1.98	(0.93-4.24)	0.078			
Cirrhosis (yes vs no)	2.23	(1.26-3.98)	0.006	2.25	(1.26-4.03)	0.006	1.59	(1.08-2.37)	0.020	1.69	(1.13-2.52)	0.010
AFP (≥400 vs <400) (ng/ml)	1.13	(0.71-1.78)	0.607				1.32	(0.92-1.87)	0.129			
Tumor diameter (>5 vs ≤5) (cm)	2.52	(1.63-3.89)	<0.001	2.23	(1.42-3.50)	<0.001	1.82	(1.30-2.53)	<0.001	1.85	(1.31-2.60)	<0.001
Tumor number (multiple vs solitary)	1.67	(0.95-2.93)	0.072				1.68	(1.09-2.59)	0.019	1.83	(1.18-2.85)	0.007
MVI (yes vs no)	2.67	(1.67-4.27)	<0.001	2.25	(1.37-3.67)	0.001	1.50	(1.07-2.11)	0.018	1.42	(1.01-2.01)	0.045
Tumor capsule (complete vs incomplete)	0.56	(0.31-1.00)	0.051				0.90	(0.61-1.32)	0.583			

HBeAg, hepatitis B e antigen; HBV, hepatitis B virus; T2DM, type 2 diabetes mellitus; RBC, red blood cells; WBC, white blood cells; HGB, hemoglobin; PLT, platelets; TBil, total bilirubin; ALB, albumin; ALT, alanine aminotransferase; AST, aspartate aminotransferase; PT, prothrombin time; AFP, alpha-fetoprotein; MVI, microvascular invasion.

### Intraoperative indicators and postoperative short-term outcomes

As shown in **Table 3**, length of hospital stay was significantly shorter in HCC patients without T2DM than those with T2DM (*P* < 0.001). In addition, the postoperative complication rates of liver failure (*P* = 0.019), ascites (*P* = 0.007), pulmonary or abdominal infection (*P* = 0.016), and surgical site infection (SSI) (*P* = 0.02) were significantly lower in patients without T2DM compared to those with T2DM. Pringle’s maneuver and its occlusion time, anatomical resection, major hepatectomy, surgical margin, blood loss amount, transfusion, operation time, incidence of bile leakage and pleural effusion were comparable between the two groups.

**Table 3:** Intraoperative variables and Postoperative short-term outcomes of all HCC patients (n=402).

Characteristics	Without T2DM (n=340)	With T2DM (n=62)	*P* value
**Intraoperative variables**			
Pringle’s maneuver	299 (87.9%)	56 (90.3%)	0.591
Duration of occlusion (min)	23.1 (20.3-29.5)	25.4 (22.3-31.8)	0.475
Anatomical resection	156 (45.9%)	24 (38.7%)	0.296
Major hepatectomy	101 (29.7%)	17 (27.4%)	0.832
Surgical margin >1 (cm)	220 (64.7%)	35 (56.5%)	0.272
Blood loss (ml)	180 (100-375)	230 (200-495)	0.051
Transfusion	26 (7.7%)	7 (11.3%)	0.478
Operation time (min)	167 (115-230)	173 (135-226)	0.523
**Postoperative outcomes**			
Hospitalization (days)	8.2 (5.5-12.5)	12.3 (9.2-16.0)	<0.001
Complications			
Liver failure	8 (2.4%)	5 (8.1%)	0.019
Bile leakage	21 (6.2%)	5 (8.1%)	0.578
Pleural effusion	6 (1.8%)	3 (4.8%)	0.148
Ascites	51 (15%)	18 (29%)	0.007
Pulmonary/Abdominal infection	12 (3.5%)	7 (11.3%)	0.016
Surgical site infection	11 (3.2%)	6 (9.7%)	0.020

### Association of T2DM with survival in HCC patients undergoing LLR

**Figure 2:**
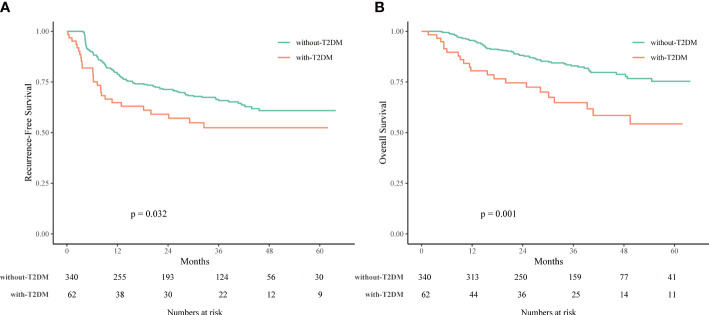
Recurrence-free survival (RFS) **(A)** and overall survival (OS) **(B)** of HCC patients associated with or without type 2 diabetes mellitus (T2DM) treated with laparoscopic liver resection (LLR).

As shown in **Figure 2**, the T2DM group had significantly worse RFS and OS rates compared to the non-T2DM group (*P* = 0.032 and 0.001, respectively). The 1-, 3-, and 5-year RFS rates were 64.8%, 52.4%, and 52.4%, respectively, for patients with T2DM, and 78.8%, 65.8%, and 60.9%, respectively, for patients without T2DM (**Supplementary Table 1**). The 1-, 3-, and 5-year OS rates were 80.5%, 64.8%, and 54.3%, respectively, for patients with T2DM, and 95.5%, 82.9%, and 75.3%, respectively, for patients without T2DM (**Supplementary Table 1**).

### T2DM was an independent risk factor associated with incidence of MVI

As shown in **Supplementary Table 2**, we found that co-existing T2DM (*P* = 0.027) was an independent risk factor associated with incidence of MVI, along with HBV DNA load > 10^4^ IU/mL (*P* = 0.003), no antiviral treatment (*P* = 0.003), AFP ≥ 400 ng/mL (*P* = 0.006), tumor diameter > 5 cm (*P* = 0.001), incomplete tumor capsule (*P* = 0.004), and cirrhosis (*P* = 0.026).

### Association of T2DM with survival in HCC patients with MVI

The clinicopathological characteristics of HCC patients with MVI are shown in **Supplementary Table 3**. HCC patients with MVI and concomitant T2DM were significantly older (*P* = 0.008), had significantly lower HBV-DNA load (*P* = 0.049), had markedly higher fasting plasma glucose level (*P* < 0.001) and markedly elevated serum creatinine level (*P* = 0.026). As shown in **Supplementary Figure 1**, RFS and OS rates were significantly worse in HCC patients who had MVI and T2DM compared to those who had MVI but did not have T2DM (both *P* = 0.002). The 1-, 3-, and 5-year RFS rates were 57.1%, 41.1%, and 41.1%, respectively, for patients with MVI and T2DM, and 75.7%, 65.5%, and 60.9%, respectively, for patients with MVI but without T2DM (**Supplementary Table 4**). The 1-, 3-, and 5-year OS rates were 75.5%, 56.5%, and 33.3%, respectively, for patients with MVI and T2DM, and 93.6%, 74.8%, and 69.8%, respectively, for patients with MVI but without T2DM (**Supplementary Table 4**).

### Additional treatments for recurrence of HCC patients after LLR

As shown in **Supplementary Table 5**, a total of 162 (40.2%) HCC patients after radical laparoscopic hepatectomy had tumor recurrence during the follow-up period. 21 (5.2%) patients underwent repeated surgery once diagnosed tumor recurrence. 29 (7.2%) patients received transarterial chemoembolization (TACE), 25 (6.2%) patients received radiofrequency ablation (RFA), and three (0.7%) patients received atezolizumab plus bevacizumab (T +A therapy). Two (0.5%) patients received the best supportive care for tumor recurrence due to liver function deterioration. 82 (20.4%) patients’ data on treatment for tumor recurrence were not available.

## Discussion

T2DM is a common chronic metabolic disease and is now estimated to affect 4%–5% of the worldwide population (8). T2DM is a chronic condition characterized by hyperglycemia, hyperinsulinemia, and insulin resistance. There are accumulating epidemiological studies showing that T2DM increases the incidence of HCC among individuals with chronic hepatitis B or C virus infection (11, 32). As the global incidences of T2DM and HCC are on the rise, a substantial proportion of patients will be affected by the two diseases.

Recently, LLR is gradually replacing traditional open hepatectomy, and has become the mainstream surgical method for HCC due to its advantages of minimal invasiveness (15, 16). In several OLR study series, preoperative T2DM has been reported to be associated with an increased risk of postoperative morbidities (17, 33) and have a direct negative impact on survival of HCC (34–36). While LLR is gaining a rapid popularity among liver surgeons, however, little is known regarding the downstream effect of T2DM after such an approach. Understanding the impact of preoperative T2DM on perioperative and survival outcomes following LLR is crucial to facilitate its adoption, as this surgical approach is expected to declare itself as the preferred technique over the traditional open method. To the best of our knowledge, this study is the first of its kind in terms of the subject.

In the present study, 15.4% of HCC patients undergoing LLR were diagnosed as diabetic preoperatively. Serum creatinine level was significantly higher in the T2DM group, in which diabetic nephropathy and impaired glomerulus function may be the causative factor. Interestingly, the proportion of MVI was found to be remarkably higher in the T2DM group compared with the non-T2DM group. Preoperative T2DM was found to be an independent risk factor for incidence of MVI. Microangiopathy is a common complication in diabetic patients. The pathophysiological changes of diabetic microangiopathy include damage of endothelial cells and thickening of basement membrane, which lead to incompleteness and fragility of the microvascular wall. Additionally, the involved microvessels may be partially or completely blocked as the progression of diabetes, contributing to the formation of hypoxia microenviroment and micro-metastasis (8). The above reasons could be reasonable explanations for the close relationship between T2DM and MVI in HCC patients undergoing laparoscopic surgery.

Given the detrimental effect that diabetes has throughout the body, it has conventionally been considered that these patients are at a higher risk of postoperative complications. Not surprisingly, in our study, significantly prolonged hospitalization and a higher incidence of complications were noticed in the diabetic patients than their counterparts. Hyperglycemia and insulin resistance in diabetic patients may accelerate the progression of liver fibrosis, which dramatically increases the risk of posthepatectomy liver failure (37, 38). Diabetic nephropathy and protein synthesis dysfunction of liver lead to hypoproteinemia, which may become the main causative factor for higher incidence of ascites in the T2DM group. Diabetic patients tend to have sustained oxidative stress and disordered inflammation and immunity function (39, 40); diabetic patients are more vulnerable to space organ and surgical site infections due to worse physical status after surgery.

In terms of the long-term oncological survival outcomes, we demonstrated the negative influence of preoperative T2DM on HCC recurrence and overall survival following laparoscopic hepatectomy. Furthermore, we also found HCC patients with MVI and T2DM had significantly worse OS and RFS rates compared to patients with MVI but without T2DM. These results suggest that preoperative T2DM is closely linked to the prognosis of HCC patients treated with LLR regardless of MVI. These findings derived from laparoscopic surgical approach are consistent with one previous study reported by our team (41).

Several limitations of this study should be considered. First, our data did not have information regarding the duration and severity of T2DM at the time of diagnosis; therefore, the potential impact of diabetes-associated complications like cardiovascular events on survival cannot be evaluated. Second, the enrolled patients all came from China and most of them had a background of HBV infection. Whether the results can be extrapolated to other geographic areas with predominantly HCV-related HCC needs further study. Third, since metformin has been reported to have the function to reduce mortality and improve survival for diabetic patients with HCC (42, 43), the perioperative outcomes and long-term survival of diabetic HCC patients following LLR may be influenced by these hypoglycemic medications. Hence, further prospective studies are needed to explore in greater detail the prognostic significance of T2DM in HCC patients who receiving LLR.

## Conclusion

Taken together, the findings of the present study demonstrated that T2DM had a negative impact on the perioperative complications and the long-term survival outcomes of patients with HCC who underwent laparoscopic liver resection. Additionally, our study revealed a close relationship between pre-existing T2DM and the incidence of microvascular invasion in HCC based on the laparoscopic resection patient cohort. In the future, a prospective controlled trial is recommended to validate the impact of T2DM on HCC recurrence and overall outcome after laparoscopic hepatectomy.

## Data availability statement

The original contributions presented in the study are included in the article/**Supplementary Material**. Further inquiries can be directed to the corresponding authors.

## Ethics statement

The studies involving human participants were reviewed andapproved by the Institutional Ethics Committees of Eastern Hepatobiliary Surgery Hospital (EHBH), Fujian Provincial Hospital (FPH), Changzhou People’s Hospital (CZPH), and Affiliated Tumor Hospital of Guangxi Medical University (ATHGMU). The patients/participants provided their written informed consent to participate in this study.

## Author contributions

Conception and design: W-XG, S-QC, S-YY, M-LY, Y-FD, J-KF. Administrative support: W-XG, S-QC. Provision of study materials or patients: W-XG, S-QC, M-LY, Y-FD, J-ZY, JS. Collection and assembly of data: S-YY, LG, JX. Data analysis and interpretation: S-YY, J-KF, Z-HL. Statistical analysis: S-YY, J-KF, Z-HL. All authors contributed to the article and approved the submitted version.

## Supplementary material

Supplementary Figure 1 Recurrence-free survival (RFS) **(A)** and overall survival (OS) **(B)** of HCC patients with microvascular invasion (MVI) associated with or without type 2 diabetes mellitus (T2DM) treated with laparoscopic liver resection (LLR).
