# Supplementary figures and images for: Impact of type 2 diabetes mellitus on the prognosis of patients with hepatocellular carcinoma after laparoscopic liver resection: A multicenter retrospective study
Source: Front Oncol. 2022 Dec 15;12:979434. doi: 10.3389/fonc.2022.979434 (PMC9798278; doi:10.3389/fonc.2022.979434)

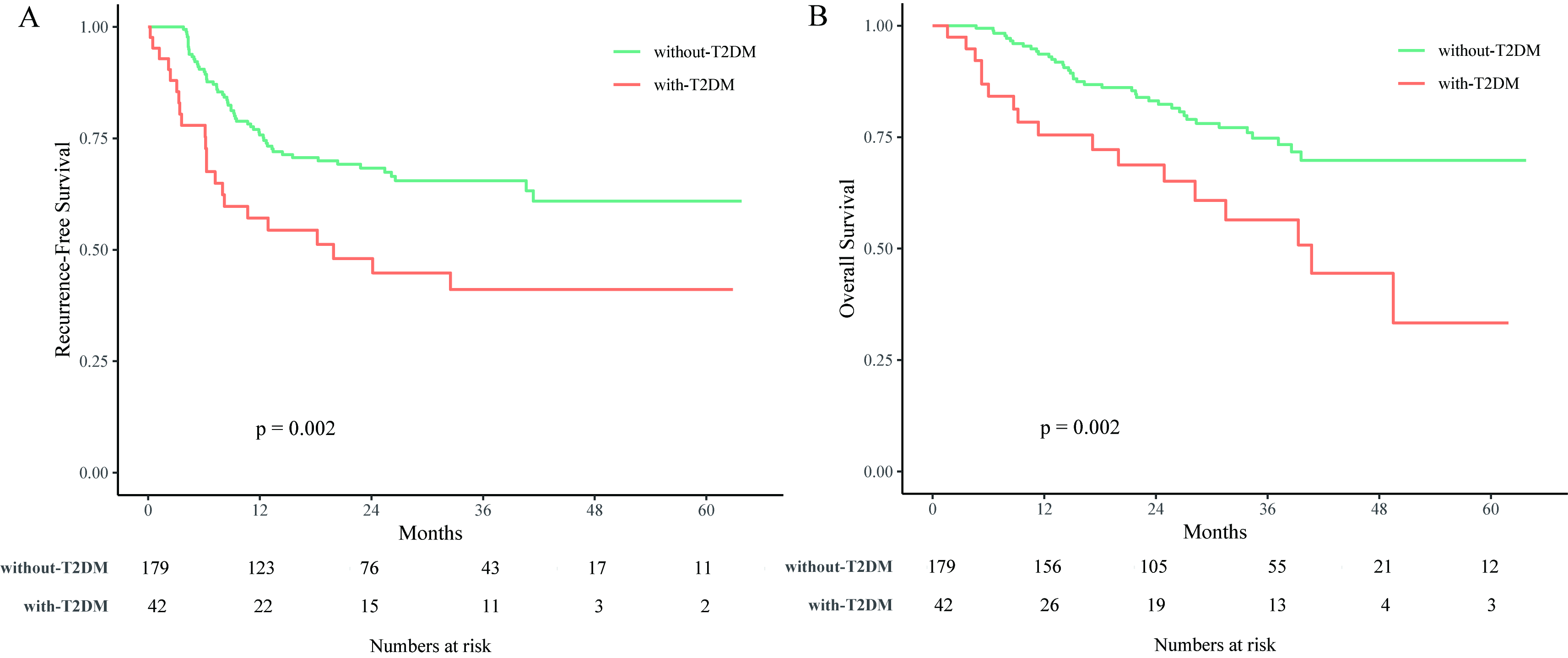

Supplement: Supplementary Figure 1 — Recurrence-free survival (RFS) (A) and overall survival (OS) (B) of HCC patients with microvascular invasion (MVI) associated with or without type 2 diabetes mellitus (T2DM) treated with laparoscopic liver resection (LLR). [file Image_1.tif]
