# Supplementary material for: Impact of type 2 diabetes mellitus on the prognosis of patients with hepatocellular carcinoma after laparoscopic liver resection: A multicenter retrospective study
Source: Front Oncol. 2022 Dec 15;12:979434. doi: 10.3389/fonc.2022.979434 (PMC9798278; doi:10.3389/fonc.2022.979434)
Supplement: Supplementary file 2 [file Table_1.docx]

**Table S1. Comparison of RFS and OS rates among all HCC patients with or without preoperative T2DM (n=402)**

| Indexes | n | 1-year | 3-year | 5-year | Log-rank | P value |
| --- | --- | --- | --- | --- | --- | --- |
| OS |  |  |  |  |  |  |
| Without T2DM | 340 | 95.5 (93.3 - 97.8) | 82.9 (78.7 - 87.4) | 75.3 (69.2 - 81.9) | 11.6 | 0.001 |
| With T2DM | 62 | 80.5 (70.8 - 91.6) | 64.8 (52.7 - 79.8) | 54.3 (40.6 - 72.7) |  |  |
| RFS |  |  |  |  |  |  |
| Without T2DM | 340 | 78.8 (74.5 - 83.3) | 65.8 (60.6 - 71.4) | 60.9 (54.9 - 67.5) | 4.6 | 0.032 |
| With T2DM | 62 | 64.8 (53.8 - 78.2) | 52.4 (40.7 - 67.6) | 52.4 (40.7 - 67.6) |  |  |

**Abbreviations:** RFS, recurrence-free survival; OS, overall survival; T2DM, type 2 diabetes mellitus.
