# Supplementary material for: Impact of type 2 diabetes mellitus on the prognosis of patients with hepatocellular carcinoma after laparoscopic liver resection: A multicenter retrospective study
Source: Front Oncol. 2022 Dec 15;12:979434. doi: 10.3389/fonc.2022.979434 (PMC9798278; doi:10.3389/fonc.2022.979434)
Supplement: Supplementary file 3 [file Table_2.docx]

Table S2 Univariate and multivariate logistic regression analysis for the incidence of MVI in HCC patients (n = 402)

| **Variables** | **Univariate analysis** | | | **Multivariate analysis** | | |
| --- | --- | --- | --- | --- | --- | --- |
|  | **HR** | **95% CI** | ***P* value** | **HR** | **95% CI** | ***P* value** |
| Age, years | 0.99 | 0.98-1.01 | 0.535 |  |  |  |
| Sex |  |  |  |  |  |  |
| Female |  |  |  |  |  |  |
| Male | 1.03 | 0.61-1.73 | 0.918 |  |  |  |
| T2DM |  |  |  |  |  |  |
| No |  |  |  |  |  |  |
| Yes | 1.89 | 1.06-3.35 | 0.030 | 2.04 | 1.09-3.82 | 0.027 |
| HBeAg |  |  |  |  |  |  |
| Negative |  |  |  |  |  |  |
| Positive | 1.20 | 0.75-1.94 | 0.459 |  |  |  |
| HBV DNA load, IU/mL |  |  |  |  |  |  |
| ≤10^4^ |  |  |  |  |  |  |
| ＞10^4^ | 2.33 | 1.39-3.40 | 0.001 | 2.37 | 1.33-4.21 | 0.003 |
| Antiviral therapy |  |  |  |  |  |  |
| No |  |  |  |  |  |  |
| Yes | 0.64 | 0.42-0.98 | 0.038 | 0.48 | 0.30-0.78 | 0.003 |
| AFP, ng/mL |  |  |  |  |  |  |
| ≥400 |  |  |  |  |  |  |
| <400 | 0.51 | 0.34-0.77 | 0.001 | 0.54 | 0.35-0.84 | 0.006 |
| ALT, U/L |  |  |  |  |  |  |
| ≤44 |  |  |  |  |  |  |
| >44 | 1.22 | 0.79-1.89 | 0.358 |  |  |  |
| AST, U/L |  |  |  |  |  |  |
| ≤44 |  |  |  |  |  |  |
| >44 | 1.15 | 0.72-1.85 | 0.563 |  |  |  |
| TBil, μmol/L |  |  |  |  |  |  |
| <17.1 |  |  |  |  |  |  |
| ≥17.1 | 1.26 | 0.81-2.03 | 0.486 |  |  |  |
| ALB, g/L |  |  |  |  |  |  |
| <35 |  |  |  |  |  |  |
| ≥35 | 0.53 | 0.23-1.12 | 0.107 |  |  |  |
| PT, s |  |  |  |  |  |  |
| ≤13 |  |  |  |  |  |  |
| >13 | 1.01 | 0.67-1.52 | 0.977 |  |  |  |
| PLT, x10^9^/L |  |  |  |  |  |  |
| ≤100 |  |  |  |  |  |  |
| >100 | 0.73 | 0.37-1.39 | 0.342 |  |  |  |
| Glucose, mmol/L |  |  |  |  |  |  |
| ≤7 |  |  |  |  |  |  |
| >7 | 1.45 | 0.74-2.97 | 0.289 |  |  |  |
| Creatinine, mg/dL |  |  |  |  |  |  |
| ≤1.2 |  |  |  |  |  |  |
| >1.2 | 1.15 | 0.36-3.95 | 0.812 |  |  |  |
| WBC, x10^6^/L |  |  |  |  |  |  |
| <4000 |  |  |  |  |  |  |
| ≥4000 | 0.81 | 0.42-1.55 | 0.532 |  |  |  |
| RBC, x10^12^/L | 0.89 | 0.65-1.23 | 0.492 |  |  |  |
| HGB, g/L |  |  |  |  |  |  |
| ≤110 |  |  |  |  |  |  |
| >110 | 0.74 | 0.29-1.80 | 0.513 |  |  |  |
| Varices |  |  |  |  |  |  |
| Present |  |  |  |  |  |  |
| Absent | 4.69 | 1.24-30.6 | 0.046 | 3.05 | 0.72-21.30 | 0.200 |
| Tumor diameter, cm |  |  |  |  |  |  |
| ≤5 |  |  |  |  |  |  |
| >5 | 1.82 | 1.19-2.78 | 0.006 | 2.17 | 1.36-3.45 | 0.001 |
| Tumor number |  |  |  |  |  |  |
| Solitary |  |  |  |  |  |  |
| Multiple | 1.39 | 0.76-2.59 | 0.287 |  |  |  |
| Tumor capsule |  |  |  |  |  |  |
| Incomplete |  |  |  |  |  |  |
| Complete | 0.42 | 0.26-0.68 | <0.001 | 0.47 | 0.28-0.78 | 0.004 |
| Cirrhosis |  |  |  |  |  |  |
| No |  |  |  |  |  |  |
| Yes | 1.74 | 1.11-2.70 | 0.015 | 1.72 | 1.07-2.77 | 0.026 |

Abbreviations: T2DM, type 2 diabetes mellitus; AFP, alpha-fetoprotein; ALT, alanine aminotransferase; AST, aspartate transaminase; HBeAg, hepatitis B e antigen; HBV, hepatitis B virus; MVI, microvascular invasion; TBil, total bilirubin; ALB, albumin; PT, prothrombin time; RBC, red blood cells; WBC, white blood cells; HGB, hemoglobin; PLT: platelets.
