# Supplementary material for: Impact of type 2 diabetes mellitus on the prognosis of patients with hepatocellular carcinoma after laparoscopic liver resection: A multicenter retrospective study
Source: Front Oncol. 2022 Dec 15;12:979434. doi: 10.3389/fonc.2022.979434 (PMC9798278; doi:10.3389/fonc.2022.979434)
Supplement: Supplementary file 4 [file Table_3.docx]

**Table** **S3** The baseline clinicopathological features of HCC patients with MVI (n=221)

| Variables | Without T2DM n=179 | With T2DM  n=42 | *P* value |
| --- | --- | --- | --- |
| Age, years | 55.4±11.5 | 60.7±11.1 | 0.008 |
| Sex |  |  | 0.257 |
| Female | 27 (15.1%) | 10 (23.8%) |  |
| Male | 152 (84.9%) | 32 (76.2%) |  |
| HBeAg |  |  | 1.000 |
| Negative | 137 (76.5%) | 32 (76.2%) |  |
| Positive | 42 (23.5%) | 10 (23.8%) |  |
| HBV DNA load, IU/mL |  |  | 0.049 |
| ≤10^4^ | 43 (24.0%) | 17 (40.5%) |  |
| ＞10^4^ | 136 (76.0%) | 25 (59.5%) |  |
| Antiviral therapy |  |  | 0.384 |
| No | 108 (60.3%) | 29 (69.0%) |  |
| Yes | 71 (39.7%) | 13 (31.0%) |  |
| AFP, ng/mL |  |  | 0.624 |
| <400 | 97 (54.2%) | 21 (50.0%) |  |
| ≥400 | 82 (45.8%) | 21 (50.0%) |  |
| ALT, U/L |  |  | 0.170 |
| ≤44 | 128 (71.5%) | 35 (83.3%) |  |
| >44 | 51 (28.5%) | 7 (16.7%) |  |
| AST, U/L |  |  | 0.528 |
| ≤44 | 134 (74.9%) | 34 (81.0%) |  |
| >44 | 45 (25.1%) | 8 (19.0%) |  |
| TBil, μmol/L |  |  | 0.133 |
| <17.1 | 160 (89.4%) | 34 (81.0%) |  |
| ≥17.1 | 19 (10.6%) | 8 (19.0%) |  |
| ALB, g/L |  |  | 0.774 |
| <35 | 19 (10.6%) | 3 (7.14%) |  |
| ≥35 | 160 (89.4%) | 39 (92.9%) |  |
| PT, s |  |  | 0.735 |
| ≤13 | 69 (38.5%) | 18 (42.9%) |  |
| >13 | 110 (61.5%) | 24 (57.1%) |  |
| PLT, *10^9^/L |  |  | 0.596 |
| ≤100 | 20 (11.2%) | 6 (14.3%) |  |
| >100 | 159 (88.8%) | 36 (85.7%) |  |
| Glucose, mmol/L |  |  | <0.001 |
| ≤7 | 170 (95.0%) | 27 (64.3%) |  |
| >7 | 9 (5.03%) | 15 (35.7%) |  |
| Creatinine, mg/dL |  |  | 0.026 |
| ≤1.2 | 176 (98.3%) | 38 (90.5%) |  |
| >1.2 | 3 (1.68%) | 4 (9.52%) |  |
| WBC, *10^6^/L |  |  | 1.000 |
| <4000 | 20 (11.2%) | 5 (11.9%) |  |
| ≥4000 | 159 (88.8%) | 37 (88.1%) |  |
| RBC, *10^12^/L | 4.61 (4.23-5.02) | 4.51 (4.22-4.82) | 0.429 |
| HGB, g/L |  |  | 0.717 |
| ≤110 | 10 (5.59%) | 3 (7.14%) |  |
| >110 | 169 (94.4%) | 39 (92.9%) |  |
| Child-Pugh class |  |  | 0.416 |
| A | 161 (89.9%) | 36 (85.7%) |  |
| B | 18 (10.1%) | 6 (14.3%) |  |
| BCLC stage |  |  | 0.117 |
| 0-A | 128 (71.5%) | 35 (83.3%) |  |
| B | 51 (28.5%) | 7 (16.7%) |  |
| Varices |  |  | 0.695 |
| Absent | 169 (94.4%) | 41 (97.6%) |  |
| Present | 10 (5.59%) | 1 (2.38%) |  |
| Tumor diameter, cm |  |  | 0.058 |
| ≤5 | 101 (56.4%) | 31 (73.8%) |  |
| >5 | 78 (43.6%) | 11 (26.2%) |  |
| Tumor number |  |  | 1.000 |
| Solitary | 154 (86.0%) | 36 (85.7%) |  |
| Multiple | 25 (14.0%) | 6 (14.3%) |  |
| Tumor capsule |  |  | 0.465 |
| Complete | 26 (14.5%) | 8 (19.0%) |  |
| Incomplete | 153 (85.5%) | 34 (81.0%) |  |
| Cirrhosis |  |  | 1.000 |
| No | 62 (34.6%) | 14 (33.3%) |  |
| Yes | 117 (65.4%) | 28 (66.7%) |  |

Abbreviations: T2DM, type 2 diabetes mellitus; AFP, alpha-fetoprotein; ALT, alanine aminotransferase; AST, aspartate transaminase; HBeAg, hepatitis B e antigen; HBV, hepatitis B virus; MVI, microvascular invasion; TBil, total bilirubin; ALB, albumin; PT, prothrombin time; RBC, red blood cells; WBC, white blood cells; HGB, hemoglobin; PLT: platelets; BCLC, Barcelona Clinic Liver Cancer.
