# Supplementary material for: Impact of type 2 diabetes mellitus on the prognosis of patients with hepatocellular carcinoma after laparoscopic liver resection: A multicenter retrospective study
Source: Front Oncol. 2022 Dec 15;12:979434. doi: 10.3389/fonc.2022.979434 (PMC9798278; doi:10.3389/fonc.2022.979434)
Supplement: Supplementary file 5 [file Table_4.docx]

**Table S4 Comparison of RFS and OS rates among HCC patients with MVI with or without preoperative T2DM (n=221)**

| Indexes | n | 1-year | 3-year | 5-year | Log-rank | P value |
| --- | --- | --- | --- | --- | --- | --- |
| OS |  |  |  |  |  |  |
| Without T2DM | 179 | 93.6 (90.0 – 97.3) | 74.8 (67.7 – 82.6) | 69.8 (61.5 – 79.2) | 9.8 | 0.002 |
| With T2DM | 42 | 75.5 (62.7 – 90.8) | 56.5 (41.0 – 77.7) | 33.3 (16.1 – 69.2) |  |  |
| RFS |  |  |  |  |  |  |
| Without T2DM | 179 | 75.7 (69.6 – 82.4) | 65.5 (58.4 – 73.5) | 60.9 (52.3 – 71.0) | 9.2 | 0.002 |
| With T2DM | 42 | 57.1 (43.6 – 74.9) | 41.1 (27.5 – 61.3) | 41.1 (27.5 – 61.3) |  |  |

**Abbreviations:** RFS, recurrence-free survival; OS, overall survival; MVI, microvascular invasion; T2DM, type 2 diabetes mellitus.
