# Supplementary material for: Impact of type 2 diabetes mellitus on the prognosis of patients with hepatocellular carcinoma after laparoscopic liver resection: A multicenter retrospective study
Source: Front Oncol. 2022 Dec 15;12:979434. doi: 10.3389/fonc.2022.979434 (PMC9798278; doi:10.3389/fonc.2022.979434)
Supplement: Supplementary file 6 [file Table_5.docx]

Table S5 Additional treatments for recurrence of patients after laparoscopic liver resection.

|  | All patients (n = 402) |
| --- | --- |
| RS | 21 (5.2%) |
| TACE | 29 (7.2%) |
| RFA | 25 (6.2%) |
| T+A | 3 (0.7%) |
| BSC | 2 (0.5%) |
| NA | 82 (20.4%) |

RS, repeated surgery; TACE, transarterial chemoembolization; RFA, radiofrequency ablation; T+A, atezolizumab combined with bevacizumab; BSC, best supportive care; NA, data not available.
